# Characteristics and Formula Test of Protein‐Based Products From *Belontia hasselti* Wetlands in South Kalimantan

**DOI:** 10.1155/ijfo/6411610

**Published:** 2026-11-01

**Authors:** Hesty Heryani, Pardi Affandi, Hastirullah Fitrah, Riza Adrianoor Saputra, Agung Cahyo Legowo

**Affiliations:** ^1^ Department of Agro-industrial Technology Faculty of Agriculture University of Lambung Mangkurat Banjarbaru Indonesia ulm.ac.id; ^2^ Department of Mathematics Faculty of Mathematics and Natural Sciences University of Lambung Mangkurat Banjarbaru Indonesia ulm.ac.id; ^3^ Department of Agribusiness Faculty of Agriculture University of Achmad Yani Banjarbaru Indonesia; ^4^ Department of Agroecotechnology Faculty of Agriculture University of Lambung Mangkurat Banjarbaru Indonesia ulm.ac.id

**Keywords:** air fryer, *Belontia hasselti*, choline, MBG, RP-HPLC, SEM–EDX

## Abstract

The utilization of locally available food resources in the Indonesian Free Nutritious Meals (MBG) program has the potential to shorten supply chains, improve raw material freshness, enhance nutritional quality, and stimulate regional economic development. This study is aimed at (1) analyzing and characterizing the raw materials used in the formulation; and (2) developing selected formulations emphasizing the nutritional value of local resources as a potential strategy to support the Indonesian Free Nutritious Meals (MBG) program. Five prototype products were initially developed from the freshwater fish *Belontia hasselti*, followed by sensory evaluation to select the most promising prototype. The selected prototype was subsequently optimized by evaluating five formulations prepared from different local fish species through sensory analysis, reverse‐phase high‐performance liquid chromatography (RP‐HPLC), scanning electron microscopy coupled with energy‐dispersive x‐ray spectroscopy (SEM–EDX), recommended dietary allowance (RDA) assessment, and economic feasibility analysis. Among the evaluated formulations, Formula Kr (*B. hasselti*) showed consistently favorable sensory acceptance across the evaluated attributes and was selected for subsequent characterization based on its relevance to the study objective and local wetland resource utilization. Formula Kr also exhibited the highest choline content (121.84 mg/100 g dry weight). SEM analysis revealed relatively continuous and compact surface regions with fewer prominent surface voids in Kr compared with the more irregular and discontinuous surface observed in Ml. EDX analysis identified carbon and oxygen as the major elements in both formulations. Based on a standard serving size of 69 g, Formula Kr provided 84.07 mg of choline, contributing up to 33.63% of the Indonesian RDA for children aged 7–12 years. Economic analysis indicated favorable production feasibility with an R/C ratio of 1.35. These findings indicate that Nemobite Formula Kr possesses favorable nutritional, physicochemical, sensory, and economic characteristics, highlighting its potential as a locally sourced functional food to support the Indonesian Free Nutritious Meals (MBG) program.

## 1. Introduction

Food security is a major global concern that underpins the availability of sufficient, safe, and nutritious food, including in Indonesia. Food Law No. 18 of 2012 defines food security as the availability of affordable food in sufficient quantity, quality, nutrition, and safety, and does not violate religious/belief requirements or customs. This condition enables individuals to live productively and maintain optimal health in a sustainable manner. A country′s food security index is based on four indicators: food availability, affordability, quality and safety, and sustainability [1, 2].

Globally, free school meal programs have proven to be an effective tool for combating malnutrition, increasing school enrollment, and reducing socioeconomic disparities. Large‐scale multisectoral initiatives in Brazil, India, and Finland have demonstrated how structured feeding programs, integrated into education and health systems, can improve child nutrition, increase school attendance, and generate local economic impact [3, 4]. In Indonesia, the Free Nutritious Meals (MBG) program aims to improve nutritional status while strengthening local food systems through the utilization of regional food resources [5]. Increasing the use of nutrient‐rich local foods is considered an effective strategy to support food security, improve dietary quality, and stimulate local agricultural development [4, 6, 7]. The program forms part of Indonesia′s long‐term strategy to develop a healthy generation by 2045 [8, 9].

To support programs like MBG effectively, optimizing local natural resources is essential. Banjar Regency, with its extensive wetland ecosystems and river basins, offers considerable potential as a source of locally available food resources [10, 11]. One prominent local commodity is the freshwater fish *Belontia hasselti*, which is highly adaptable to its environment and serves as a sustainable source of animal protein [12, 13].

Adequate intake of high‐quality protein and essential micronutrients is important for normal growth and development, particularly during childhood and pregnancy. Among these nutrients, choline plays an essential role in cell membrane synthesis and normal neurological development, whereas folate contributes to DNA synthesis and cell division [14, 15]. To enhance the nutritional and functional characteristics of the proposed formulation, locally available functional plants were incorporated into the formulation. *Origanum vulgare* contains phenolic compounds, including carvacrol and thymol, whereas *Clitoria ternatea* is rich in flavonoids and anthocyanins with antioxidant properties [16–19].

Despite the high nutritional and functional potential of these local wetland resources, their application in standardized functional food formulations remains limited. Developing scientifically based formulations from locally available ingredients is important to improve nutritional quality and increase the utilization of regional food resources. Therefore, this study is aimed at (1) analyzing and characterizing the ingredients used in the formulation; and (2) developing selected formulations emphasizing the nutritional value of local resources as a potential strategy to support the Indonesian Free Nutritious Meals (MBG) program.

## 2. Materials and Methods

### 2.1. Materials and Formula Preparation

Fish flour from *B. hasselti* was prepared using a Philips 2000 Series Airfryer (Model NA230/00; Philips, Amsterdam, The Netherlands; manufactured in China), with a 6.2‐L capacity, 1700 W power rating, and RapidAir technology. The air fryer employs rapid hot‐air circulation to dehydrate and cook the material with minimal oil. This processing method was selected because previous studies have reported its suitability for producing dried food products while reducing oil usage during processing [20].

Functional ingredients, including *O. vulgare*, *C. ternatea* L., and selected spices, were dried (or lightly fried in the case of garlic) before use to reduce their moisture content and facilitate subsequent processing.

Butterfly pea flower powder was prepared by separating the flowers from the stems and selecting only fresh blue flowers. The flowers were washed with running water and dried in a food dryer at 50°C–55°C for approximately 24 h before being ground and sieved through a 40‐mesh sieve.

### 2.2. Formulation Design of Prototypes

This study was conducted in two sequential stages. Stage I focused on the development and sensory screening of five prototype functional food products based on *B. hasselti*. The most promising prototype was subsequently selected for further optimization. In Stage II, the selected prototype was reformulated using five different local fish species and subjected to physicochemical, nutritional, and economic analyses. The overall study workflow is illustrated in Figure 1.

**Figure 1:**
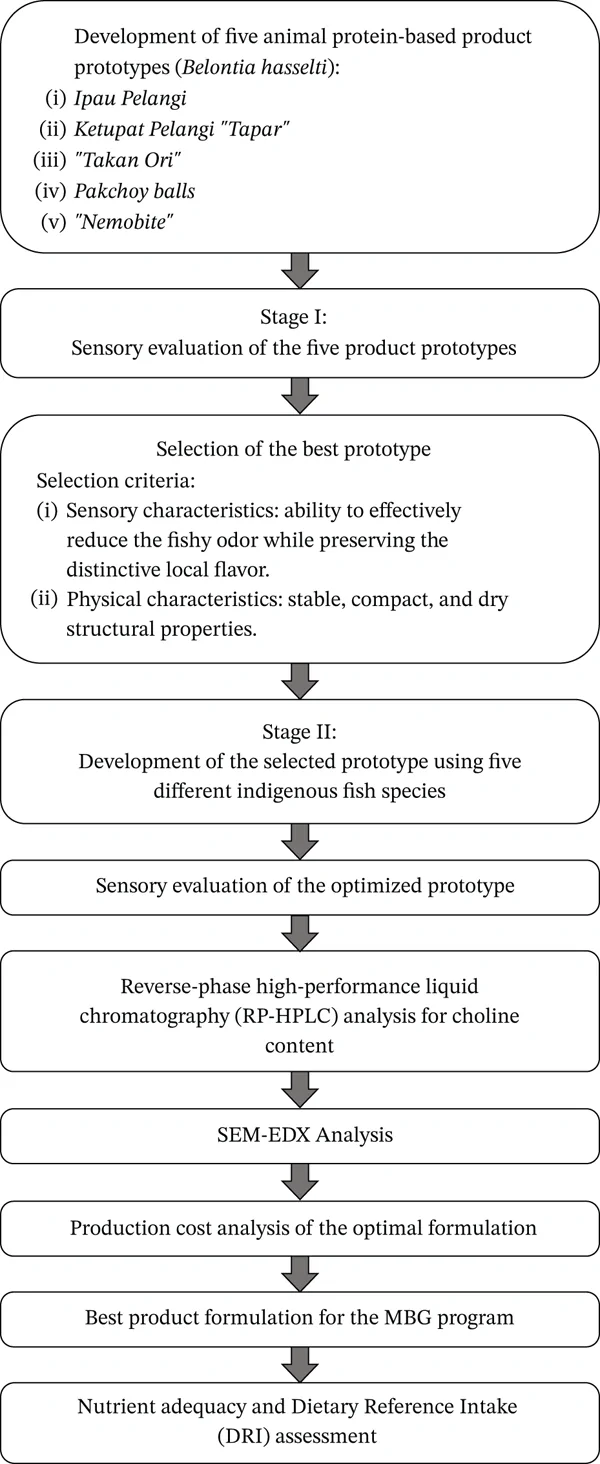
Flowchart of the development of functional food products based on local resources from South Kalimantan.

#### 2.2.1. Stage I: Development of Prototype Products

In the first stage, five prototype functional food products were developed using *B. hasselti* as the primary animal protein source, combined with locally available functional ingredients from South Kalimantan. This preliminary stage is aimed at identifying the most suitable food prototype for further formulation and characterization based on consumer acceptance and product characteristics.

The five prototypes developed were Ipau Pelangi, Ketupat Pelangi “Tapar,” Takan Ori, Pakchoy Balls, and Nemobite. Each prototype was formulated with different combinations of local ingredients to enhance nutritional quality, sensory characteristics, and product attractiveness while maintaining the utilization of regional food resources. A summary of the prototype formulations is presented in Table 1.

**Table 1:** Summary of prototype formulations.

Prototype	Primary protein source	Key functional ingredients	Development objective
Ipau Pelangi	*Belontia hasselti*	Pakcoy and broccoli, *Clitoria ternatea* L., dragon fruit, carrots, *Origanum vulgare*	Evaluate consumer acceptance of multilayer functional food
Ketupat Pelangi “Tapar”	*Belontia hasselti*	Teak leaf and dragon fruit, *C. ternatea* L. and melon, carrot and papaya	Develop mineral‐rich colored staple food
“Takan Ori”	*Belontia hasselti*	Pumpkin flour and wheat flour (1:3), *O. vulgare*	Increase choline and folate content
Pakchoy balls	*Belontia hasselti*	Pakchoy leaf extract, *C. ternatea* L., *O. vulgare*, garlic	Incorporate leafy vegetables into fish product
“Nemobite”	*Belontia hasselti*	*O. vulgare*, standard spices	Develop child‐friendly functional snack

*Note:* Table 1 summarizes the five functional food prototypes developed during Stage I before sensory screening. The principal ingredients and development objectives of each prototype are summarized in Table 1.

All prototypes were subjected to sensory preference evaluation involving 30 untrained consumer panelists. The sensory attributes assessed included color, aroma, texture, and taste using a 5‐point hedonic scale. The use of untrained consumer panelists was considered appropriate because the sensory evaluation was conducted under real consumption conditions to measure consumer preference rather than descriptive sensory characteristics. Consumer panelists represent the intended target market and are commonly employed in hedonic acceptance tests to evaluate product acceptability [21]. A panel size of 30 participants was considered adequate for this preliminary consumer acceptance screening [22], as the primary objective of Stage I was to identify the most preferred prototype for further optimization rather than to estimate consumer preferences at the population level. The results of the sensory evaluation, together with observations of product physical characteristics, were used to select the most promising prototype for further optimization in Stage II. The selection criteria included overall sensory acceptability, the ability to reduce the characteristic fishy odor without compromising the local flavor profile, and the production of a stable dry product suitable for subsequent physicochemical characterization.

#### 2.2.2. Stage II: Optimization of the Selected Prototype

Following the preliminary sensory screening in Stage I, Nemobite was selected as the prototype for further optimization based on its favorable sensory acceptance and physical characteristics. The selected prototype demonstrated the ability to reduce the characteristic fishy odor while maintaining the distinctive local flavor and producing a stable dry structure suitable for subsequent physicochemical analyses.

In Stage II, the Nemobite formulation was optimized by evaluating five different locally available fish species as the primary protein source while maintaining identical nonprotein ingredients, processing conditions, and formulation procedures. The evaluated formulations were coded as Kr (*Belontia hasselti*), Ml (*Sardinella lemuru*), Da (*Rastrelliger kanagurta*), Tn (*Pangasius hypophthalmus*), and Hr (*Channa striata*). A summary of the formulation codes is presented in Table 2. The selected Nemobite prototype after Stage I is shown in Figure 2.

**Table 2:** Summary of Nemobite formulations evaluated in Stage II.

Formulation code	Fish species	Scientific name	Purpose
Kr	Kapar	*Belontia hasselti*	Reference/local wetland fish
Ml	Lemuru	*Sardinella lemuru*	Comparative formulation
Tn	Patin	*Pangasius hypophthalmus*	Comparative formulation
Da	Kembung	*Rastrelliger kanagurta*	Comparative formulation
Hr	Haruan	*Channa striata*	Comparative formulation

**Figure 2:**
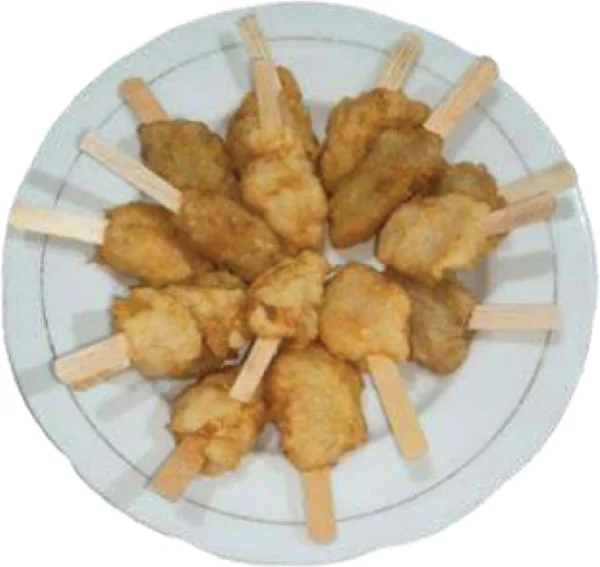
Selected Nemobite prototype after Stage I.

### 2.3. HPLC Analysis

The second stage of formulation selection employed reverse‐phase high‐performance liquid chromatography (RP‐HPLC) to quantify the choline content retained in five Nemobite formulations prepared from different local fish species. Choline analysis was performed according to the RP‐HPLC method described by Mateeva et al. [23], using an Agilent 1260 Infinity II HPLC system equipped with a 1260 Infinity II DAD WR (Wide Range) detector (Agilent Technologies, Santa Clara, California, United States).

Prior to chromatographic analysis, samples were prepared by solid–liquid extraction followed by solid‐phase extraction (SPE) using a Thermo Scientific SOLA SCX cartridge (10 mg/1 mL; Thermo Fisher Scientific, United States) under vacuum conditions to isolate choline from the food matrix.

Chromatographic separation was carried out on a Purospher C18 column (5 *μ*m, 250 × 4.6 mm; Merck KGaA, Darmstadt, Germany) under isocratic conditions using acetonitrile and water (75:25, v/v) as the mobile phase at a flow rate of 1.0 mL/min with an injection volume of 0.5 *μ*L.

Choline concentrations were quantified using an external calibration curve prepared from choline standards at concentrations ranging from 10 to 100 *μ*g/mL. The calibration curve was generated by plotting chromatographic peak area (*y*) against choline concentration (*x*), and showed excellent linearity with a coefficient of determination (*R* ^2^) of 0.9999. The concentration of choline in each sample solution was calculated from the resulting regression equation based on the chromatographic peak area obtained from the integrator.

Formulation‐specific extraction volumes and dilution factors were used during sample preparation. The HPLC‐derived choline concentration was subsequently normalized to dry matter by accounting for the extraction volume, dilution factor, and dry‐matter weight of the analyzed sample.

The choline concentration was calculated using the following equation:

(1) CDM=C×V×DFWDM,

where *C* is the choline concentration obtained from the regression equation based on the HPLC chromatographic peak area (*μ*g/mL), *V* is the extraction volume (mL), *D* *F* is the dilution factor, and *W*_*D* *M*_ is the dry‐matter weight of the analyzed sample (g). The resulting value was expressed as *μ*g/g dry matter and subsequently converted to mg/100 g dry weight. The formulation‐specific parameters and calculations are presented in Table S8.

Each formulation was analyzed once (single determination) by RP‐HPLC. Therefore, the reported choline concentrations should be interpreted as comparative analytical values among formulations rather than estimates of analytical variability.

The selected formulation was subsequently evaluated for its contribution to the Indonesian Recommended Dietary Allowance (RDA). A standard serving size of 69 g was used for the nutritional contribution calculation, with the serving‐size framework for processed foods referenced to BPOM Regulation No. 26 of 2021 [24] using the following equation:

(2) Choline content per serving 69 g=Choline concentration100×69 g.

The percentage contribution to the daily RDA was calculated based on the Indonesian Ministry of Health Regulation No. 28 of 2019 [25] using the following equation:

(3) %RDA contribution=Choline content per servingRDA standards for the target group ×100%.

### 2.4. Scanning Electron Microscopy Coupled With Energy‐Dispersive X‐Ray Spectroscopy (SEM‐EDX) Analysis

The surface morphology and elemental composition of the Kr and Ml Nemobite formulations were characterized using SEM–EDX using a ZEISS EVO 10 scanning electron microscope (Carl Zeiss Microscopy GmbH, Germany), following previously reported food material characterization methods [26, 27]. Powder samples were mounted on double‐sided carbon tape and coated with a thin conductive gold layer to minimize charging during observation. The samples were examined under high‐vacuum conditions using an accelerating voltage of 10–15 kV at a magnification of 500×. Quantitative elemental analysis was performed using the EDX detector on the same observation area to determine the elemental composition of the sample [28].

### 2.5. Cost Calculation and Profit Margin

The economic feasibility of the selected Nemobite formulation was evaluated through production cost analysis, including total production cost (TC), cost of goods sold (COGS), selling price, total revenue (TR), profit (*π*), net profit margin (NPM), break‐even point (BEP), and revenue‐to‐cost ratio (R/C ratio). The calculations were performed using established agroindustrial economic evaluation methods reported in previous studies [29–32]. Detailed definitions of variables and calculation procedures are provided in Table S6.

Production costs consisted of fixed and variable costs incurred during product manufacturing. Economic feasibility was assessed using NPM, BEP, and R/C ratio to evaluate the commercial potential of the selected formulation. The calculation procedures followed the methods described by previous studies [31–34] without modification.

NPM was calculated to evaluate profit efficiency [33] using Equation (4):

(4) NPM=πTR×100%.

The BEP was calculated for both production units and selling price [34] using Equations (5) and (6):

(5) BEP unit=TFCP−VC,

(6) BEP price=TCQ.

Business feasibility was assessed using the R/C ratio [34] using Equation (7):

(7) RC=TRTC.

An R/C ratio greater than 1 indicates that the business is economically feasible, whereas a value equal to 1 indicates the BEP, and a value below 1 indicates that the business is economically unfeasible [34].

### 2.6. Statistical Analysis

Sensory evaluation data obtained from 30 untrained consumer panelists were analyzed using repeated‐measures analysis of variance (ANOVA), as each panelist evaluated all five Nemobite formulations. Data are presented as mean ± standard deviation (SD). The assumption of sphericity was assessed using Mauchly′s test. When the assumption of sphericity was violated, the Greenhouse–Geisser correction was applied to adjust the degrees of freedom and *p* values. When significant differences among formulations were detected (*p* < 0.05), Duncan′s multiple range test (DMRT) was applied for multiple comparisons. Different superscript letters indicate statistically significant differences among formulations within each sensory attribute at the 5% significance level. Statistical analyses were performed using a significance threshold of *p* < 0.05 (Table 3). Detailed ANOVA results are provided in Tables S1, S2, S3, and S4.

**Table 3:** Mean (± SD) sensory preference scores of the five Nemobite formulations based on a 5‐point hedonic scale.

Formulation	Color	Aroma	Texture	Taste
Kr	3.733 ± 0.785^a^	3.700 ± 0.915^a^	3.300 ± 0.877^abc^	3.867 ± 0.900^a^
Ml	2.967 ± 1.098^b^	3.167 ± 1.234^b^	3.100 ± 1.062^bc^	3.267 ± 0.944^b^
Tn	3.800 ± 0.761^a^	3.867 ± 0.819^a^	3.633 ± 0.765^a^	3.900 ± 0.960^a^
Da	3.400 ± 1.003^ab^	3.600 ± 0.770^ab^	3.033 ± 0.999^c^	3.633 ± 0.928^ab^
Hr	3.800 ± 0.847^a^	3.933 ± 1.015^a^	3.533 ± 0.937^ab^	3.900 ± 0.885^a^

*Note:* Values are presented as mean ± standard deviation (*n* = 30). Within each sensory attribute (column), means with different superscript letters differ significantly according to Duncan′s multiple range test (DMRT; *p* < 0.05), whereas means sharing at least one superscript letter are not significantly different at the 5% significance level. Sample codes represent the primary fish protein utilized: Kr (*Belontia hasselti*), Ml (*Sardinella lemuru*), Tn (*Pangasius hypophthalmus*), Da (*Rastrelliger kanagurta*), and Hr (*Channa striata*).

Quantitative data obtained from RP‐HPLC analysis are presented descriptively because the objective was to compare normalized choline concentrations among the evaluated formulations rather than to perform inferential statistical analysis. SEM–EDX observations were interpreted descriptively based on representative micrographs and elemental composition. Economic feasibility indicators (COGS, NPM, BEP, and R/C ratio) were calculated using established agroindustrial economic equations without inferential statistical testing.

## 3. Results and Discussion

The Free Nutritious Meals (MBG) program is fundamentally linked to the green economy, encouraging local consumption, waste reduction, and community empowerment. These elements are key indicators in the Green Economy Index (GEI), which assesses sustainability through resource efficiency, emission reduction, food security, and local economic empowerment [33, 34]. This study is aimed at developing a food product that perfectly aligned with these indicators by utilizing local wetland resources.

The study consisted of two sequential stages. Stage I screened five different product prototypes, whereas Stage II optimized the selected prototype (Nemobite) using five different fish species.

In Stage I, five traditional functional food prototypes based on *Belontia hasselti* were successfully developed: Ipau Pelangi, Ketupat Pelangi “Tapar,” Takan Ori, Pakchoy Balls, and Nemobite. Following the preliminary consumer preference evaluation, Nemobite was selected for further optimization because it effectively reduced the characteristic fishy odor while preserving the local taste profile. The selected prototype also exhibited a stable dry structure and low water activity (< 0.6), characteristics that may contribute to improved storage stability; however, shelf‐life evaluation was beyond the scope of the present study.

### 3.1. Sensory Evaluation of Nemobite Formulations

Following the selection of Nemobite in Stage I, five fish‐based formulations were evaluated to identify the optimal formulation for subsequent physicochemical and nutritional characterization. Sensory evaluation was conducted to compare the acceptability of the five Nemobite formulations (Kr, Ml, Tn, Da, and Hr) among 30 untrained consumer panelists. Four sensory attributes, namely color, aroma, texture, and taste, were assessed using a 5‐point hedonic scale. Repeated‐measures ANOVA revealed significant differences among formulations for all evaluated sensory attributes (color, aroma, texture, and taste) (*p* < 0.05). Detailed statistical results are presented in Tables S1, S2, S3, and S4.

Color: Tn, Hr, and Kr showed similarly high color preference scores, whereas Ml exhibited the lowest visual acceptance.

Aroma: Hr, Tn, and Kr showed the highest aroma preference scores, whereas Da exhibited an intermediate level of acceptance (ab) and Ml received the lowest score (b).

Texture: Significant differences were observed among formulations (*p* < 0.05). Tn exhibited the highest texture preference score (3.63, superscript a), followed by Hr (ab). Kr and Ml showed intermediate acceptance (abc and bc, respectively), whereas Da received the lowest texture preference score (c).

Taste: Kr, Tn, and Hr exhibited similarly high taste preference scores, whereas Ml showed the lowest taste acceptance. Da showed an intermediate level of acceptance.

Overall formulation selection: Although Tn and Hr obtained the highest mean scores for several sensory attributes, Kr (*Belontia hasselti*) consistently showed good acceptability across all attributes and was selected because it represented the target wetland fish species investigated in this study. Furthermore, the use of *Belontia hasselti* aligns with the study objective of developing a functional food based on locally available wetland resources for the Indonesian Free Nutritious Meals (MBG) program. Therefore, Kr was selected for subsequent physicochemical characterization, nutritional evaluation, and economic feasibility analysis.

### 3.2. Chemical and Microscopic Characterization

Quantification of choline in the five Nemobite formulations was performed using RP‐HPLC with UV detection at 205–208 nm. The external calibration curve demonstrated excellent linearity, with the regression equation y = 5.01x − 0.12 and a coefficient of determination (*R* ^2^ = 0.9999), where *y* represents the chromatographic peak area and *x* represents the choline concentration (*μ*g/mL). The retention times of all formulations ranged from 3.490 to 3.572 min, indicating consistent chromatographic performance (Table 4). After normalization on a dry‐weight basis, Formula Kr (*Belontia hasselti*) exhibited the highest choline content at 121.84 mg/100 g dry weight, whereas the remaining formulations contained lower concentrations.

**Table 4:** RP‐HPLC results and choline content of the five Nemobite formulations.

Formulation result code	Retention time (min)	Area of the integrator (*y*)	Solution concentration (*x*) (mg/L)	Choline content (mg/100 g dry weight)
Kr	3.538	406.8111	81.22	121.84
Ml	3.490	409.2129	81.70	102.13
Tn	3.524	219.3742	43.81	98.58
Da	3.512	1856.1028	370.50	92.63
Hr	3.572	155.7975	31.12	46.68

*Note:* Sample codes represent the primary fish protein utilized: Kr (*Belontia hasselti*), Ml (*Sardinella lemuru*), Tn (*Pangasius hypophthalmus*), Da (*Rastrelliger kanagurta*), and Hr (*Channa striata*).

Although Formula Da produced the largest chromatographic peak area, normalization revealed considerably lower choline content than Formula Kr. This finding indicates that chromatographic peak area alone was insufficient to compare choline content among formulations because differences in sample matrices affected the analytical response. Similar observations have been reported in studies of complex food matrices, where normalization is necessary for reliable comparison of choline concentrations among different formulations [35].

SEM examination revealed qualitative differences in surface morphology between the Kr and Ml formulations (Figure 3). The Kr formulation showed relatively continuous and compact surface regions with fewer prominent surface voids, whereas the Ml formulation exhibited a more irregular and discontinuous surface with visible depressions, voids, and fissure‐like features. These observations were based on qualitative visual assessment of the representative SEM micrographs at 500× magnification.

**Figure 3:**
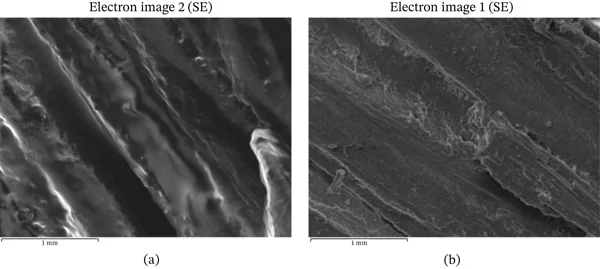
SEM surface morphology of (a) Kr (D2K5) and (b) Ml (D2M3) formulations.

EDX analysis complemented the SEM observations. The Kr formulation (D2K5) was predominantly composed of carbon (86.92 wt%) and oxygen (12.76 wt%), whereas the Ml formulation (D2M3) contained carbon (66.28 wt%), oxygen (17.42 wt%), and nitrogen (12.82 wt%), together with detectable Na, K, P, S, and Cl (Figures 4 and 5). Thus, compared with Kr, Ml showed a lower relative carbon content and higher relative oxygen and nitrogen contents. These differences in elemental composition provided complementary information on the distinct characteristics of the two formulations.

**Figure 4:**
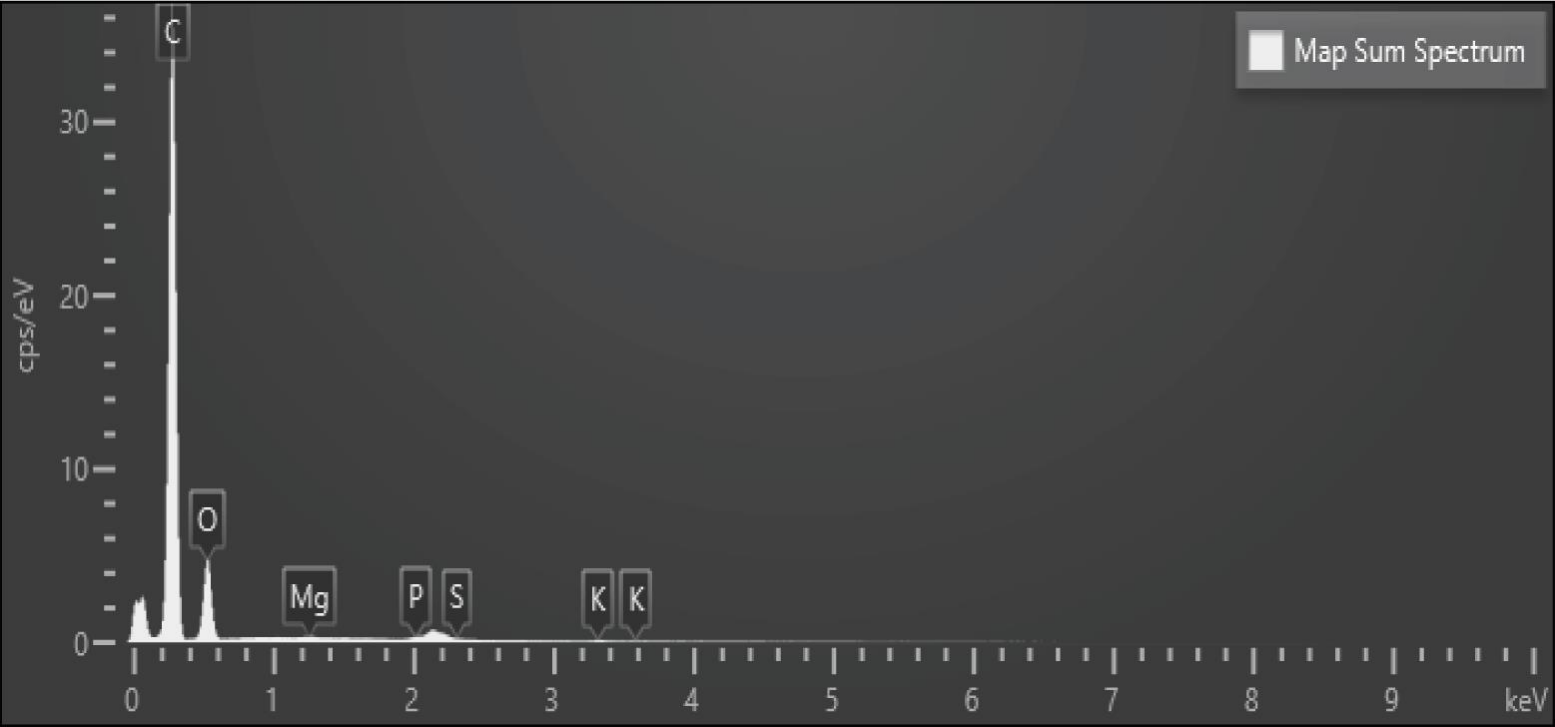
EDX spectrum of the Kr (D2K5) formulation.

**Figure 5:**
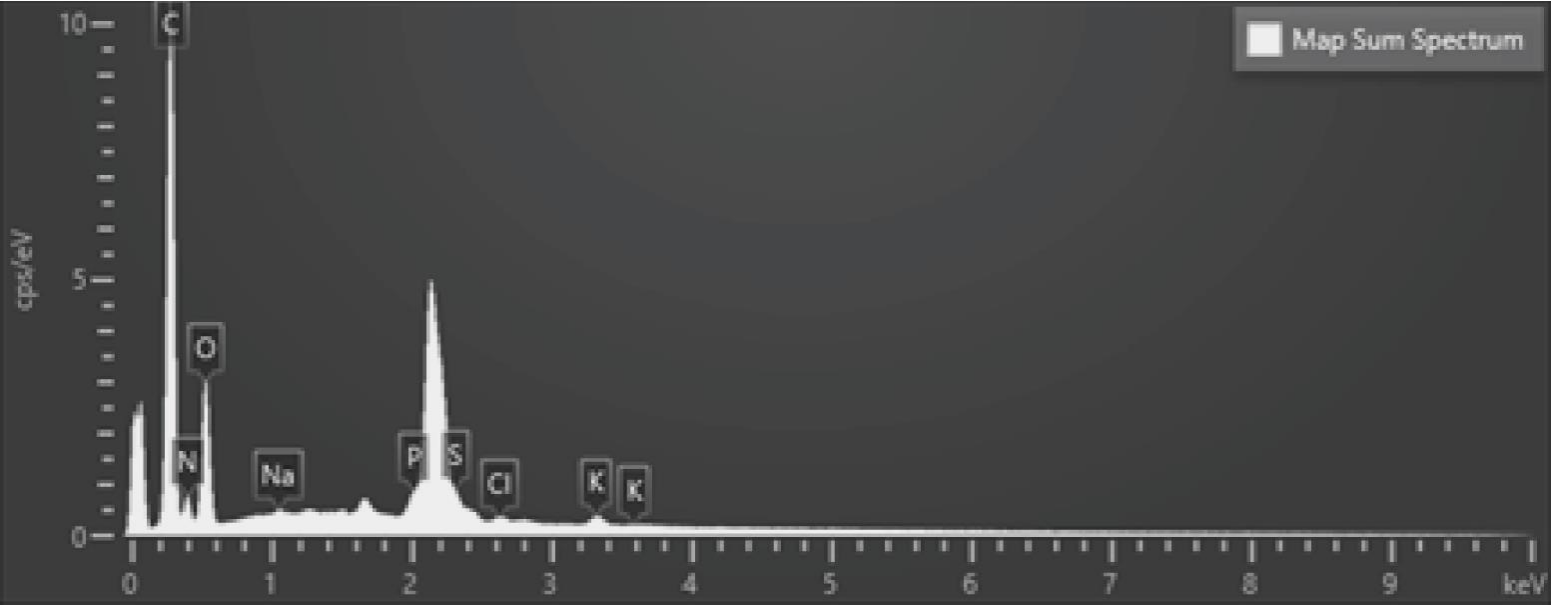
EDX spectrum of the Ml (D2M3) formulation.

The relatively continuous and less porous appearance of the Kr surface may indicate greater structural compactness than that observed in Ml. However, because no quantitative image analysis or storage experiment was performed, this interpretation remains qualitative and does not establish differences in long‐term storage stability or nutrient retention.

### 3.3. Contribution of Nemobite Kr to the RDA

The nutritional contribution of the selected Nemobite Kr formulation was evaluated by comparing its choline content with the Indonesian RDA established in the Regulation of the Minister of Health of the Republic of Indonesia No. 28 of 2019. Based on RP‐HPLC analysis (Table 4), Formula Kr exhibited the highest choline content, at 121.84 mg/100 g dry weight. For the nutritional contribution assessment, one serving was defined as three pieces of Nemobite Kr, with each piece weighing 23 g on a dry‐weight basis, resulting in a total serving size of 69 g dry weight. Based on the measured choline concentration, one 69‐g dry‐weight serving provided 84.07 mg of choline. The estimated contribution of this amount to the RDA for different target groups is presented in Table 5.

**Table 5:** Choline content and contribution to the Recommended Dietary Allowance (RDA) of Nemobite Kr per serving (69 g dry weight; three pieces × 23 g).

Nutritional parameters	Unit	Per 100 g	Per serving (69 g)	Target group	National RDA (mg/day)	Contribution to RDA (%)
Choline	mg	121.84	84.07	Elementary school student (7–12)	250.00	33.63
				Junior high school student (13–15)	375.00	22.42
				Senior high school student (16–18)	400.00	21.02
				Pregnant women (second and third trimesters)	450.00	18.68

As shown in Table 5, one serving of Nemobite Kr (69 g dry weight, equivalent to three pieces) provided 84.07 mg of choline, corresponding to 33.63%, 22.42%, 21.02%, and 18.68% of the daily choline requirement for elementary school children aged 7–12 years, junior high school students aged 13–15 years, senior high school students aged 16–18 years, and pregnant women in the second and third trimesters, respectively.

Choline is an essential nutrient involved in normal neurological function, membrane phospholipid synthesis, and methyl‐group metabolism. Adequate dietary choline intake has been associated with normal growth and development, particularly during childhood and pregnancy. Therefore, the relatively high choline contribution provided by Nemobite Kr suggests its potential as a complementary food product to support the nutritional objectives of the Indonesian Free Nutritious Meals (MBG) program. However, the present study evaluated only the choline content of the product, and no clinical assessment of health outcomes was performed.

The nutritional contribution across various age groups demonstrated that the Nemobite Formula Kr was highly practical and effective as a supplementary food product. Its relatively high choline content suggests that the product has potential as a supplementary food to help improve dietary choline intake among the target populations. The product may serve as a complementary food option to support the nutritional objectives of the Indonesian Free Nutritious Meals (MBG) program, particularly among school‐age children and pregnant women.

Compared with commonly consumed dietary sources of choline, Nemobite Kr provides an alternative locally sourced supplementary food with substantial choline content while offering good sensory acceptability [36]. These characteristics support its potential use as a locally available complementary food within the Indonesian MBG program.

### 3.4. Economic Feasibility: COGS and Profit Margin

The economic feasibility of Nemobite was evaluated to determine its commercial potential for household‐ and SME‐scale production within the Indonesian Free Nutritious Meals (MBG) program. Production costs consisted of fixed costs (equipment depreciation) and variable costs (raw materials, utilities, and labor). Detailed cost components are presented in Tables S5 and S6. Based on these analyses, the TC for one production cycle was IDR 2,550,000, comprising IDR 689,400 in fixed costs and IDR 1,860,600 in variable costs.

As summarized in Table 6, the calculated COGS was IDR 2602 per unit, whereas the product selling price was established at IDR 3500 per unit using a cost‐plus pricing approach. Under these assumptions, one production cycle generated a TR of IDR 3,430,000 and a net profit of IDR 880,000.

**Table 6:** Economic feasibility indicators for Nemobite production.

Indicator	Value
Total production cost	IDR 2,550,000
COGS	IDR 2602/unit
Product selling price	IDR 3500/unit
Total revenue	IDR 3,430,000
Net profit	IDR 880,000
Net profit margin	25.66%
Break‐even point	431 units
Break‐even price	IDR 2602/unit
R/C ratio	1.35

Financial feasibility analysis yielded a NPM of 25.66%, a BEP of 431 units, and an R/C ratio of 1.35. Since the selling price exceeded the COGS and the R/C ratio was greater than one, Nemobite production can be considered economically feasible for small‐scale commercial production.

These findings are consistent with those reported by Samanta et al. [37], who found that value‐added fish products generated positive economic returns with an R/C ratio of 1.25. The slightly higher R/C ratio obtained in the present study (1.35) suggests that Nemobite has favorable economic potential as a locally produced supplementary food while simultaneously increasing the added value of *Belontia hasselti*.

## 4. Conclusion

Based on the material characterization and formulation development, this study successfully developed Nemobite Formula Kr, an innovative functional food utilizing the local wetland fish *Belontia hasselti* from Banjar Regency. The selected formulation effectively reduced the characteristic fishy odor while maintaining the distinctive local flavor and exhibited low water activity (< 0.6), which may contribute to improved storage stability; however, shelf‐life evaluation was beyond the scope of this study. SEM–EDX analysis revealed relatively continuous and compact surface regions in Formula Kr, with fewer prominent surface voids than observed in Ml, whereas RP‐HPLC analysis showed that Kr had the highest choline content among the evaluated formulations (121.84 mg/100 g dry weight).

Based on a standard serving size of 69 g, Nemobite Formula Kr provided 84.07 mg of choline, contributing 33.63%, 22.42%, 21.02%, and 18.68% of the Indonesian RDA for children, adolescents, and pregnant women, respectively. These findings suggest that Nemobite has potential as a locally sourced supplementary food to support the nutritional objectives of the Indonesian Free Nutritious Meals (MBG) program. The selected formulation also demonstrated favorable consumer acceptance during sensory evaluation. Furthermore, economic analysis showed that one production cycle generated a net profit of IDR 880,000 with an R/C ratio of 1.35, indicating promising economic potential for household‐ and SME‐scale commercial production.

## Funding

This study was supported by Directorate of Research and Community Service, Directorate General of Research and Development, Ministry of Higher Education, Science, and Technology, SP DIPA‐139.04.1.693320/2025 revision 4 dated April 30, 2025.

## Conflicts of Interest

The authors declare no conflicts of interest.

## Supporting Information

Additional supporting information can be found online in the Supporting Information section.

## Supporting information


**Supporting Information 1** The supporting materials are available online and include additional statistical analyses, production cost calculations, economic equations, RP‐HPLC analytical conditions, choline normalization calculations, and representative photographs of the prototype products. Table S1 presents the repeated‐measures ANOVA results for color preference.

**Supporting Information 2** Table S2 presents the repeated‐measures ANOVA results for aroma preference, including the Greenhouse–Geisser correction for violation of sphericity.

**Supporting Information 3** Table S3 presents the repeated‐measures ANOVA results for texture preference.

**Supporting Information 4** Table S4 presents the repeated‐measures ANOVA results for taste preference.

**Supporting Information 5** Table S5 provides the detailed production cost calculation for the Nemobite formulation.

**Supporting Information 6** Table S6 defines the variables and economic equations used in the economic evaluation.

**Supporting Information 7** Table S7 summarizes the RP‐HPLC analytical conditions.

**Supporting Information 8** Table S8 presents the formulation‐specific calculations used to normalize choline concentrations to dry weight.

**Supporting Information 9** Figure S1 shows representative photographs of the five prototype products developed during Stage I product screening.

## Data Availability

The data that support the findings of this study are available from the corresponding author upon reasonable request.
