# Supplementary material for: Characteristics and Formula Test of Protein‐Based Products From Belontia hasselti Wetlands in South Kalimantan
Source: Int J Food Sci. 2026 Nov 1;2026:6411610. doi: 10.1155/ijfo/6411610 (PMC13724155; doi:10.1155/ijfo/6411610)
Supplement: Supplementary file 1 [file IJFO-2026-6411610-s001.docx]

**Supplementary Figure S1**

**Representative photographs of the five prototype products developed during Stage I product screening**

(A) Ipau Pelangi	(B) Ketupat Pelangi Tapar

(C) Takan Ori	(D) Pakchoy Balls

(E) Nemobite

Supplementary Figure S1. Representative photographs of the five prototype products developed during Stage I product screening: (A) Ipau Pelangi, (B) Ketupat Pelangi Tapar, (C) Takan Ori, (D) Pak choi Balls, and (E) Nemobite. The prototype products were evaluated by sensory analysis to identify the most suitable formulation for subsequent physicochemical, nutritional, and economic evaluations.
