# Supplementary material for: Characteristics and Formula Test of Protein‐Based Products From Belontia hasselti Wetlands in South Kalimantan
Source: Int J Food Sci. 2026 Nov 1;2026:6411610. doi: 10.1155/ijfo/6411610 (PMC13724155; doi:10.1155/ijfo/6411610)
Supplement: Supplementary file 2 [file IJFO-2026-6411610-s002.docx]

**Supplementary Table S1**

**Repeated-measures ANOVA for color preference among the five Nemobite formulations**

| Source of Variation | Sum of Squares | df | Mean Square | F-value | p-value |
| --- | --- | --- | --- | --- | --- |
| Formulation | 15.627 | 4 | 3.907 | 5.371 | < 0.001 |
| Residuals | 84.373 | 116 | 0.727 | - | - |

Note. Repeated-measures ANOVA with Type III Sum of Squares (n = 30)
