# Supplementary material for: Characteristics and Formula Test of Protein‐Based Products From Belontia hasselti Wetlands in South Kalimantan
Source: Int J Food Sci. 2026 Nov 1;2026:6411610. doi: 10.1155/ijfo/6411610 (PMC13724155; doi:10.1155/ijfo/6411610)
Supplement: Supplementary file 3 [file IJFO-2026-6411610-s003.docx]

**Supplementary Table S8**

**Calculation of Choline Content in Nemobite Formulations**

| **Formulation** | **HPLC concentration (µg/mL)** | **Extraction volume (mL)** | **Dilution factor** | **Dry-matter weight (g)** | **Choline (µg/g DM)** | **Choline (mg/100 g DW)** |
| --- | --- | --- | --- | --- | --- | --- |
| Kr | 81.22 | 3.00 | 5.00 | 1.00 | 1,218.36 | 121.84 |
| Ml | 81.70 | 2.50 | 5.00 | 1.00 | 1,021.29 | 102.13 |
| Tn | 43.81 | 4.50 | 5.00 | 1.00 | 985.75 | 98.58 |
| Da | 370.50 | 1.00 | 2.50 | 1.00 | 926.26 | 92.63 |
| Hr | 31.12 | 3.00 | 5.00 | 1.00 | 466.82 | 46.68 |

**Note.** Choline concentration on a dry-matter basis was calculated using the following equation:

$$C_{DM}= \frac{C x V x DF}{W_{DM}}$$

where $C$is the HPLC-derived choline concentration (µg/mL), $V$is the extraction volume (mL), $DF$is the dilution factor, and $W_{DM}$is the dry-matter weight of the analyzed sample (g). The resulting concentration (µg/g DM) was converted to mg/100 g dry weight (DW) by dividing by 10.
