# Supplementary material for: Characteristics and Formula Test of Protein‐Based Products From Belontia hasselti Wetlands in South Kalimantan
Source: Int J Food Sci. 2026 Nov 1;2026:6411610. doi: 10.1155/ijfo/6411610 (PMC13724155; doi:10.1155/ijfo/6411610)
Supplement: Supplementary file 4 [file IJFO-2026-6411610-s004.docx]

**Supplementary Table S7**

**Validation and Analytical Conditions of RP-HPLC Method**

| **Parameter** | **Value** |
| --- | --- |
| Instrument | Agilent 1260 Infinity II |
| Detector | DAD |
| Column | Purospher C18 |
| Mobile phase | ACN : Water (75:25) |
| Flow rate | 1 mL/min |
| Injection volume | 0.5 μL |
| Detection wavelength | 205–208 nm |
| Temprature | 25⁰C |
| Calibration range | 10–100 μg/mL |
| Regression Equation | y = 5.01x – 0.12 |
| Linearity (R²) | 0.9999 |
| Replicate analysis | Single determination (n = 1) |
