# Supplementary material for: Characteristics and Formula Test of Protein‐Based Products From Belontia hasselti Wetlands in South Kalimantan
Source: Int J Food Sci. 2026 Nov 1;2026:6411610. doi: 10.1155/ijfo/6411610 (PMC13724155; doi:10.1155/ijfo/6411610)
Supplement: Supplementary file 5 [file IJFO-2026-6411610-s005.docx]

**Supplementary Table S6**

**Definitions of variables and economic equations used in the economic evaluation of Nemobite formulation**

| **Variable** | **Definition** | **Economic equation** |
| --- | --- | --- |
| **TFC** | Total fixed cost (IDR) | Sum of annual depreciation costs of all equipment used in the production process |
| **TVC** | Total variable cost (IDR) | Sum of all variable production costs incurred during one production cycle |
| **TC** | Total production cost (IDR) | **TC = TFC + TVC** |
| **Q** | Production output (units) | Number of Nemobite packages produced per production cycle |
| **VC** | Variable cost per unit (IDR/unit) | **VC = TVC / Q** |
| **COGS** | Cost of goods sold (IDR/unit) | **COGS = TC / Q** |
| **P** | Selling price (IDR/unit) | Selling price assigned to one unit of Nemobite |
| **TR** | Total revenue (IDR) | **TR = P × Q** |
| **NP** | Net profit (IDR) | **NP = TR − TC** |
| **NPM** | Net profit margin (%) | **NPM = (NP / TR) × 100** |
| **BEP** | Break-even production quantity (units) | **BEP = TFC / (P − VC)** |
| **R/C ratio** | Revenue-cost ratio | **R/C = TR / TC** |

**Note.** Economic indicators were calculated using standard production cost analysis based on one production cycle of the optimized Nemobite formulation. Definitions and equations were adapted from established economic evaluation methods in food processing and agribusiness [31-34].
Detailed production cost data are presented in Supplementary Table S5.
