# Supplementary material for: Characteristics and Formula Test of Protein‐Based Products From Belontia hasselti Wetlands in South Kalimantan
Source: Int J Food Sci. 2026 Nov 1;2026:6411610. doi: 10.1155/ijfo/6411610 (PMC13724155; doi:10.1155/ijfo/6411610)
Supplement: Supplementary file 6 [file IJFO-2026-6411610-s006.docx]

**Supplementary Table S5**

**Detailed production cost calculation for Nemobite formulation**

| A. **Fixed costs** | | | | | | | | | |  |
| --- | --- | --- | --- | --- | --- | --- | --- | --- | --- | --- |
| **Details** | **Quantity** | | **Price/unit (IDR)** | **Total Price (IDR)** | | | **Economic life (years)** | **Annual depreciation (IDR)** | |  |
| Gas stove | 1 | | 300,000 | 300,000 | | | 5 | 54,000 | |  |
| Chopper | 2 | | 150,000 | 300,000 | | | 2 | 135,000 | |  |
| Baking pan | 2 | | 30,000 | 60,000 | | | 2 | 27,000 | |  |
| Scale | 1 | | 130,000 | 130,000 | | | 5 | 23,400 | |  |
| Steamer | 2 | | 100,000 | 200,000 | | | 2 | 90,000 | |  |
| Air fryer | 2 | | 1,000,000 | 2,000,000 | | | 5 | 360,000 | |  |
| Total fixed cost (TFC) | | | | | | | | 689,400 | |  |
| B. **Variable costs** | | | | | | | |  | |  |
| **Details** | | **Quantity** | | | **Unit** | **Price (IDR)** | | | **Total Price (IDR)** | |
| Fresh fish | | 20000 | | | g | 50 | | | 1,000,000 | |
| Healthy mushroom-based seasoning | | 1000 | | | g | 160 | | | 160,000 | |
| Oregano | | 1000 | | | g | 180 | | | 180,000 | |
| Aluminium foil roll | | 1 | | | unit | 270,000 | | | 270,000 | |
| Electricity (Air fryer operation) | | 22.4 | | | kWh | 1,500 | | | 33,600 | |
| LPG fuel | | 2 | | | unit | 33,500 | | | 67,000 | |
| Direct labor | | 3 | | | people | 50,000 | | | 150,000 | |
| Total variable cost (TVC) | | | | | | | | | 1,860,600 | |
| **Total production cost (TFC + TVC)** | | | | | | | | | **2,550,000** | |

**Note.** Fixed costs were estimated using the straight-line depreciation method based on the expected economic life of each equipment item. Variable production costs represent the principal cost components included in the economic evaluation of the optimized Nemobite formulation. Minor ingredients and consumables were incorporated into the aggregated production cost.
