# Supplementary material for: Characteristics and Formula Test of Protein‐Based Products From Belontia hasselti Wetlands in South Kalimantan
Source: Int J Food Sci. 2026 Nov 1;2026:6411610. doi: 10.1155/ijfo/6411610 (PMC13724155; doi:10.1155/ijfo/6411610)
Supplement: Supplementary file 8 [file IJFO-2026-6411610-s008.docx]

**Supplementary Table S2**

**Repeated-measures ANOVA for aroma preference among the five Nemobite formulations**

| Source of Variation | Sum of Squares | df | Mean Square | F-value | p-value |
| --- | --- | --- | --- | --- | --- |
| Formulation | 10.973 | 2.499 | 4.391 | 4.153 | 0.013 |
| Residuals | 76.627 | 72.479 | 1.057 | - | - |

Note. Repeated-measures ANOVA with Type III Sum of Squares (n = 30). Mauchly's test indicated a violation of the sphericity assumption (W = 0.342, χ²(9) = 29.391, p < 0.001). Therefore, Greenhouse–Geisser correction was applied (ε = 0.625). The corrected test indicated a significant effect of formulation on aroma preference, *F*(2.499, 72.479) = 4.153, *p* = 0.013.
