# Supplementary material for: Characteristics and Formula Test of Protein‐Based Products From Belontia hasselti Wetlands in South Kalimantan
Source: Int J Food Sci. 2026 Nov 1;2026:6411610. doi: 10.1155/ijfo/6411610 (PMC13724155; doi:10.1155/ijfo/6411610)
Supplement: Supplementary file 9 [file IJFO-2026-6411610-s009.docx]

**Supplementary Table S3**

**Repeated-measures ANOVA for texture preference among the five Nemobite formulations**

| Source of Variation | Sum of Squares | df | Mean Square | F-value | p-value |
| --- | --- | --- | --- | --- | --- |
| Formulation | 8.240 | 4 | 2.060 | 2.650 | 0.037 |
| Residuals | 90.160 | 116 | 0.777 | - | - |

Note. Repeated-measures ANOVA with Type III Sum of Squares (n = 30)
